# Severe Elephant Endotheliotropic Herpesvirus 6 Associated Disease in Two African Elephants Under Human Care in Austria

**DOI:** 10.3390/ani15101482

**Published:** 2025-05-20

**Authors:** Stella Knüppel, Folko Balfanz, Christiane Riedel, Verena Strauss, Tabitha E. Hoornweg, Katharina Dimmel, Karin Walk, Anna Kübber-Heiss, Annika Posautz, Thomas Voracek, Azza Abdelgawad, Jakob Trimpert, Stephan Hering-Hagenbeck, Till Rümenapf, Angelika Auer

**Affiliations:** 1 Department of Biological Sciences and Pathobiology, Infectiology and Virology, University of Veterinary Medicine, Veterinärplatz 1, 1210 Vienna, Austria; stella.knueppel@vetmeduni.ac.at (S.K.); christiane.riedel@ens-lyon.fr (C.R.); katharina.dimmel@vetmeduni.ac.at (K.D.); karin.walk@vetmeduni.ac.at (K.W.); till.ruemenapf@vetmeduni.ac.at (T.R.); 2 Tiergarten Schönbrunn, Maxingstraße 13 b, 1130 Vienna, Austria; f.balfanz@zoovienna.at (F.B.);; 3 Department of Interdisciplinary Life Sciences, Research Institute of Wildlife Ecology, University of Veterinary Medicine, Savoyenstraße 1, 1160 Vienna, Austria; verena.strauss@vetmeduni.ac.at (V.S.); anna.kuebber@vetmeduni.ac.at (A.K.-H.);; 4 Department of Biomolecular Health Sciences, Division of Infectious Diseases and Immunology, Faculty of Veterinary Medicine, Utrecht University, Yalelaan 1, 3584 CL Utrecht, The Netherlands; 5 Tierärztliche Ordination Tiergarten Schönbrunn, Seckendorff-Gudent-Weg 6, 1130 Vienna, Austria; t.voracek@zoodoc.at; 6 Center for Infection Medicine, Institute of Virology, School of Veterinary Medicine, Freie Univerität Berlin, Kaiserswerther Str. 16-18, 14195 Berlin, Germany; azza.abdelgawad@hhu.de (A.A.); jtrimpert@vet.k-state.edu (J.T.)

**Keywords:** *Loxodonta africana*, trunk wash, *Proboscivirus*, EEHV6, EEHV3, elephant

## Abstract

In 2021, a captive two-year-old African elephant died of cardiovascular failure in Austria. Lesions were indicative of elephant endotheliotropic herpesvirus (EEHV) associated disease, and the presence of EEHV6 could be detected by PCR. About two months later, an eight-year-old female elephant from the same herd showed typical clinical signs of EEHV-related hemorrhagic disease, which coincided with EEHV6 viremia. The animal underwent anti-herpesviral therapy and recovered quickly. Subsequently, blood and trunk wash samples from all elephants of the herd were monitored weekly for the presence of EEHV6-specific nucleic acids by qPCR. A retrospective analysis of the samples revealed repeated EEHV6 reactivation and the presence of EEHV3 in the samples of one animal. Our findings underline the threat EEHV6 poses to juvenile African elephants and emphasize the need for regular monitoring of viremia and virus excretion to prevent fatalities and predict transmission events.

## Simple Summary

In 2021, a two-year-old African elephant died of cardiovascular failure in an Austrian zoo. Elephant endotheliotropic herpes virus 6 (EEHV6) was identified as the underlying cause. About two months later, another herd member displayed symptoms of EEHV infection but survived. Subsequently, all elephants of the herd were monitored weekly for the presence of EEHVs. Further investigations revealed that EEHVs were repeatedly shed in the herd. Our findings underline the threat EEHVs pose to juvenile African elephants and emphasize the need for regular monitoring to prevent fatalities and predict transmission events.

## 1. Introduction

Elephant endotheliotropic herpesviruses (EEHVs) are a group of herpesviruses that naturally infect elephants and may cause acute, often fatal, hemorrhagic disease in young elephants. Since their discovery in 1988, hundreds of cases have been reported in Asian elephants (*Elephas maximus*) [1,2,3,4,5,6], whereas only a few cases of EEHV-associated diseases in African elephants (*Loxodonta africana*) have been documented [1,4,6,7,8,9]. Seven EEHVs are known to date. EEHV1, EEHV4 and EEHV5 are associated with Asian elephants, whilst EEHV2, EEHV3, EEHV6 and EEHV7 naturally infect African elephants [6,10,11]. EEHVs are genetically distinct from other herpesviruses and were assigned to the genus *Proboscivirus* within the *Betaherpesvirinae* subfamily [12]. In 2014, Richman et al. [13] proposed assigning EEHVs to a new subfamily, the *Deltaherpesvirinae*. EEHVs seem to have co-evolved with elephants and their predecessors for the last 100 million years [10]. Several studies demonstrated that EEHVs are frequently detectable in sample materials of healthy Asian [14,15,16] and African elephants [11,17]. In contrast to reports of EEHV hemorrhagic diseases in Asian elephants [1,2,3,18], only a few cases of clinical diseases and/or fatalities in African elephants have been documented to date [1,4,7,8,9,19]. These include a fatal case of EEHV6 in a 10-year-old male in Thailand [7], a 15-month-old calf in Arkansas that survived EEHV6 viremia after famciclovir treatment in 2009 [4], a male calf that died from EEHV2 hemorrhagic disease in California in 1996 [1], and two outbreaks of EEHV3-related hemorrhagic diseases, including two fatalities (6.5 and 7.5 years old African elephants) and four survivors in North American zoos [8,9]. There are probably more case reports that have only been presented at local specialist conferences but have not yet been published in peer-reviewed journals, such as the two EEHV7 cases described by Latimer et al. [19] in 2022.

Here, we present clinical, (histo-) pathological, virological, and serological findings of one fatal case of EEHV6 hemorrhagic disease (HD) in a captive African elephant in Europe; clinical, virological, and serological findings of a survivor of EEHV6 hemorrhagic disease (HD); and the dynamics of EEHV viremia and secretion in this specific herd during a two-year period.

## 2. Materials and Methods

### 2.1. Study Population

African elephants have been housed at Schönbrunn Zoo (Vienna Zoo, Wien, Austria) since 1870. In 2021, the herd consisted of one adult bull (E6), two adult cows (E5, E4), and three young females aged 18 (E3), 8 (E2), and 2 (E1) years. In November 2022, E6 left and was replaced by a new bull (E7) in May 2023. This decision was made in cooperation with the European Conservation Breeding Program for African Elephants. The medical history of African elephant E2 includes a broken right tusk with exposed dental pulp in August 2017, which was treated daily with povidone–iodine flushing; no further treatments or vaccinations were administered. African elephant E1 (female, 0.1) had no notable medical findings, treatments, or vaccinations.

### 2.2. Case 1 (E1)

In summer 2021, the 2-year-old female African elephant calf (E1) suddenly presented with reduced general condition, loss of appetite, mild colic, and slight salivation. The animal was treated symptomatically with Metamizol (Richter, A 4600 Wels, Austria, 10,000 mg + Boehringer, D55216 Ingelheim, Germany, 10,000 mg) and Butylscopolaminiumbromid (Boehringer, D55216 Ingelheim, Germany, 80 mg i.m.) and died three days after the onset of symptoms.

Necropsy was performed at the pathology laboratory of the Research Institute for Wildlife Ecology, Vienna.

No bacterial pathogens able to cause the observed symptoms could be isolated from the lymph node and lung samples. Blood samples and an organ pool consisting of the intestine, liver, lymph nodes, spleen, and lung were taken for virological analysis.

Samples of E1 were sent to the Institute of Virology, University of Veterinary Medicine, Vienna. A total sample volume of 100 mg of the organ pool was tissue lysed (Tissue lyser II, Qiagen, Hilden, Germany) in 1 mL sterile PBS and centrifuged at 16,000× g for three minutes. PBMCs were isolated from EDTA blood and digested with tissue lysis buffer and proteinase K (Qiagen, Hilden, Germany). Nucleic acids were isolated from 140 µL of supernatant of the organ suspension, digested PBMCs, and EDTA plasma. Nucleic acid extractions were performed employing the QIAamp Viral RNA Mini QIAcube Kit using QIAcube (Qiagen, Hilden, Germany) according to the manufacturer’s instructions.

Nucleic acid extracts were screened for Herpesviruses employing two pan-herpesvirus primer pairs published by VanDevanter et al. 1996 [20]. This assay is known to detect a broad panel of alpha-, beta-, and gamma-herpesviruses. Samples were also screened for nucleic acids of Encephalomyocarditis virus (EMCV) by RT-qPCR [21], a virus which can also lead to acute deaths in elephants. A pan-EEHV semi-nested PCR [4] targeting the polymerase gene in DNA extracts from organ pool, plasma, and PBMCs was performed. The PCR products were cleaned with PCR Kleen Spin Columns (Bio-Rad, Vienna, Austria) and sent to Eurofins Genomics AT GmbH (Vienna, Austria) for sequencing. Using the BLAST (version 2.16.1+, National Center for Biotechnology Information) database search, generated DNA sequences were compared to sequences available in GenBank.

Based on published EEHV6 polymerase gene sequence information from GenBank, an EEHV6-specific primer set for highly sensitive conventional nested PCR was designed and optimized (Table 1).

EEHV6-specific qPCR primer and probe sequences were kindly provided by Dr. Jakob Trimpert, Institute of Virology (Freie Universität Berlin, Germany). The EEHV6 qPCR’s limit of quantification was determined by running doubles of a ten-fold serial dilution of a plasmid standard and was defined as 1 × 10^5^ GE/mL for plasma and trunk wash samples and 1 × 10^6^ GE/g for organ samples. This corresponds to 2.3 × 10^2^ GE/reaction. Samples giving qPCR results below these values or questionable qPCR curves were re-tested and eventually confirmed by EEHV6-specific nested PCR.

All used PCR kits and primer/probe sequences are shown in Table 1.

### 2.3. Case 2 (E2)

On the 9th of September 2021, three months after the initial, fatal case, the eight-year-old elephant cow (E2) displayed nonspecific clinical signs concurrent with positive EEHV6 qPCR results. An immediate symptomatic and anti-herpesviral treatment was started (see the Section 3 for more details).

### 2.4. EEHV-Specific Antibody Status of the Vienna Zoo Elephant Herd

After the initial case, EEHV-specific antibody levels were determined for all elephants present at Vienna Zoo using a published multiple EEHV species gB ELISA [22,23].

### 2.5. Further Herd Monitoring and EEHV3 Detection

From the 17th of August 2021 on, trunk wash samples and EDTA blood samples from all herd members have been analyzed weekly by EEHV6 qPCR. After comparing standard dilution series and performing a retrospective analysis of positive samples, the qPCR protocol used until 2023 was replaced by a qPCR protocol for EEHV6 detection published by Stanton et al. (2012) [24] (see Table 1) due to its higher sensitivity (limit of detection at 10 copies/reaction compared to 230 copies/reaction) and higher fluorescence increase due to the MGB (minor groove binder) probe. The comparison of the two EEHV6 qPCRs is shown in the Supplementary Figure S1.

To be able to assess the occurrence of other EEHV viruses in the herd, 74 selected elephant samples (plasma and trunk wash samples) from all herd members taken at 21 different sampling dates were retrospectively analyzed for EEHV2- and EEHV3-specific DNA using published qPCR protocols [24]. Samples from all elephants that were chronologically related to the EEHV6-HD case from July 2021 were chosen. Additionally, plasma and trunk wash samples were selected over the full screening period from 2021 to 2023.

## 3. Results

### 3.1. Case 1—Pathological and Virological Findings

The carcass was well preserved and appeared to be well-nourished. Significant gross lesions in the thoracic cavity, including brownish-yellow pericardial effusion of approximately 5 liters and a severe dilatation of the right ventricle, were detected. In addition, a severe, partly coalescing ecchymosis in the epicardium expanding to the coronary fat was noted. The myocardium appeared heterogeneous, and the histopathological examination revealed degeneration of myocardial cells, severe leucocytostasis, and multifocal perivascular mixed inflammatory infiltrates.

The pleura visceralis showed massive edematization of up to 3–5 cm and was tightly adhered to the severely emphysematous pleura parietalis, partly conjoining with the diaphragm. Histological investigation of the lungs showed a severe disseminated interstitial and alveolar edema, severe multifocal–coalescing hemorrhages, and multifocally large amounts of macrophages admixed within the edema and smaller amounts of fibrin.

Furthermore, a massive disruption of the endothelia of small and large vessels in the heart, lungs, and liver was noticed during histological examination. There was a partial loss of vessel wall structures and extravasation of erythrocytes, admixed with other blood cells, into the surrounding parenchyma. These lesions were also found, but were less pronounced, in the kidneys, spleen, and lymph nodes.

Significant gross lesions observed in the abdominal cavity were an ascites consisting of 2–3 liters of a clear-yellow, mildly stringy fluid, generalized swelling of lymph nodes and spleen, and a severely swollen and acutely congested liver. Histopathology of lymph nodes showed blood resorption and edema, and active hyperplastic follicles with active germinal centers, karyorrhectic cells, cellular debris, and partially severe infiltration with macrophages. Lymphocyte depletion was observed in the spleen. Histopathology of the liver revealed scattered subacute-chronic microabscesses, severe hemorrhages due to the vessel lesions mentioned above, as well as mild to moderate multifocal degeneration of hepatocytes.

The gastrointestinal tract was well filled. A striking feature was the very large number of stones (diameter ~2–3 cm) in the large intestine. The neighboring mucosa showed disseminated hemorrhages ≤ 2 mm. The intestinal serosa and the mesentery showed massive coalescing edema with a special focus on the area of the small intestine and caecum.

Virological analysis was performed to evaluate the presence of viral pathogens able to cause corresponding clinical symptoms and pathological changes. The pan-herpesvirus nested PCR by VanDevanter et al. 1996 [20], as well as an EMCV-RT-qPCR, delivered negative results. The specific bands of 237 bp, which could be detected with a pan-EEHV semi-nested PCR [4], were sequenced and were identical to EEHV6 sequences available in the gene bank. Absolute quantification by qPCR revealed viral loads of 8 × 10^7^ GE/g organ pool sample and 5 × 10^6^ GE/mL plasma.

### 3.2. Case 2 (E2) and Subclinical Shedding in Animal E3

On the 9th of September 2021, three months after the initial, fatal case, the eight-year-old elephant cow (E2) displayed listlessness and slightly reduced general behavior, but normal appetite and cooperative training behavior. Immediate therapy with Meloxicam 0.6 mg/kg p.o. (Boehringer Ingelheim, Ingelheim/Rhein, Germany, 3750 mg) and Famciclovir 15 mg/kg BID p.o. (Phoenix Labs, County Meath, Dublin, Ireland, 10,500 mg) was started. On the following day, medical training was possible, but E2 showed reduced appetite and stiff motion. Therapy was continued with Famciclovir 15 mg TID p.o. (Phoenix Labs, County Meath, 10,500 mg). On 11th September, intensive therapy was started in standing sedation. Sedation was started with Detomidine 30 mg (0.016 mg/kg; Richter Pharma 4600 Wels 150 mg + Vetcare Limited 24101 Salo, 150 mg) and Butorphanol 30 mg (0.016 mg/kg; Richter Pharma, 4600 Wels, Austria, 150 mg) and deepened with Detomidine 10 mg (0.005 mg/kg; Richter Pharma, 4600 Wels, 150 mg + Vetcare Limited, 24101 Salo, 150 mg) and Butorphanol 50 mg (0.027 mg/kg; Richter Pharma, 4600 Wels, 150 mg) after 28 min. A plasmatransfusion of 350 mL (0.2 mL/kg) from E2’s mother was given i.v., as well as Trimetoprim/Sulfadoxin 5 mg/kg (Virbac, 06516 Carros + 1180 Wien, Austria, 20,000 mg), Flunixinmeglumin 2.2 mg/kg (Vet-Agro Trading Sp., 20–234 Lublin, Poland, 5000 mg), Metamizol/Coffein 20 mg/kg (Vetviva Richter GmbH, 4600 Wels, Austria, 1000 g), and 5 L of NaCl (0,9 %). Rectal infusion with warm water (about 100 L) was performed, Famciclovir 15 mg/kg rectal and Zylexis^®^, an inactivated parapoxvirus ovis (iPPVO) 2 mL i.m. (Zoetis GmbH, 10785 Berlin, Germany, 10 × 1 mL). Sedation was reversed with Atipamezole 250 mg (Virbac GmbH, 1080 Wien, Austria, 50 mg) and Naloxone 250 mg i.m. (Wildlife Pharmaceuticals, 1240 White River, MO, USA, 1000 mg). On September 12th, the same treatments were given under sedation. The following day, E2 was more active and less cooperative, and sedation and treatment were performed as on the previous day. After three days of intensive therapy, E2 again showed normal behavior and a good appetite. The treatment followed the recommendations of the EEHV advisory group (https://eehvinfo.org/, accessed on 5 May 2025).

**Figure 1:**
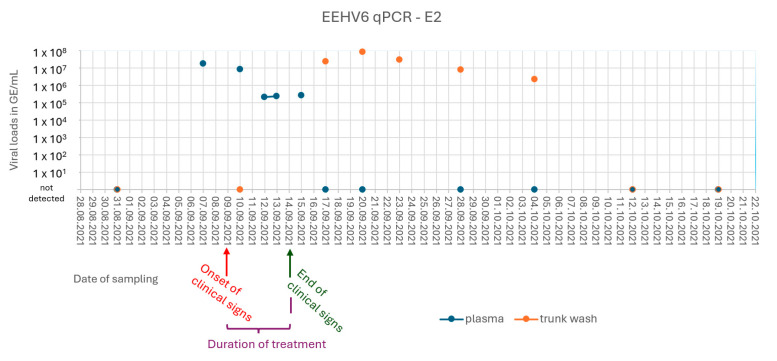
Graphical summary of E2’s EEHV6 qPCR results over time. Viral loads in genome equivalents per milliliter (GE/mL).

Plasma samples and PBMCs of E2 taken on September 7 were PCR positive for EEHV6 (1.8 × 10^7^ GE/mL plasma). Viremia lasted for nine days, and shedding from the trunk was first observed 10 days after the initial detection of EEHV6 in plasma and lasted for 18 days (Figure 1 and Supplementary Table S1).

**Figure 2:**
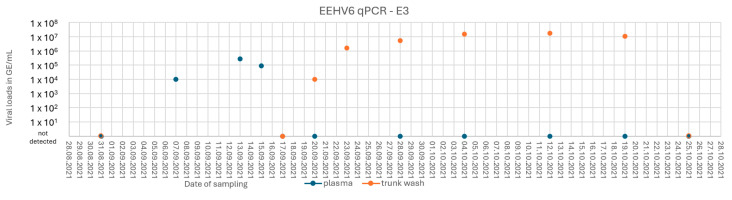
Graphical summary of E3’s EEHV6 qPCR results over time. Viral loads in genome equivalents per milliliter (GE/mL).

A clinically healthy 18-year-old elephant cow (E3) also displayed low-level viremia from the 7th to the 15th of September 2021, and a significant excretion of EEHV6 in trunk fluids over a period of at least 30 days (Figure 2 and Supplementary Table S1) could be observed.

### 3.3. EEHV-Specific Antibody Status of the Vienna Zoo Elephant Herd

**Figure 3:**
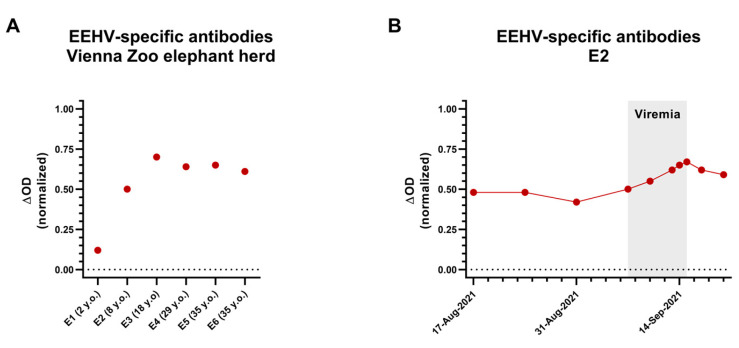
Quantification of EEHV-specific antibody levels using the multiple EEHV species-specific gB ELISA. (**A**) EEHV-specific antibodies were detected for all herd members present at the Zoo during the EEHV6 outbreak. The sample from animal E1 (case 1) was taken perimortem, while the other samples were taken approximately two months after the death of E1. (**B**) EEHV-specific antibody levels were detected for animal E2 (case 2) between 17 August 2021 and 20 September 2021. The period in which EEHV6 viremia was detected is indicated in gray.

All elephants, apart from the 2-year-old E1 (case 1), displayed distinct antibody levels to different EEHVs (Figure 3A), suggesting they were at least infected with one EEHV species. Only very low levels of EEHV-specific antibodies were detectable in a peri-mortem serum sample of E1 (case 1), which could potentially still be of maternal origin. Therefore, we assume that animal E1 was still immunologically naïve for all EEHVs before succumbing to EEHV6-HD. Animal E2 (case 2) already had detectable levels of EEHV-specific antibodies before the development of EEHV-HD-like symptoms in September 2021, suggesting that she had already been in contact with at least one EEHV species in the past (Figure 3B). EEHV6 viremia caused a clearly detectable rise in EEHV-specific antibody levels in this animal (Figure 3B).

### 3.4. Further Herd Monitoring and EEHV3 Detection

EEHV6 was again detected in trunk flush samples of E2 in October 2022. Detection was limited to two samples taken two days apart, and the viral loads detected were less than 1 × 10^5^ GE/mL trunk wash. The new male elephant (E7) introduced into the herd in 2023 also shed EEHV6 intermittently for approximately two weeks after his arrival. Viral loads were close to the limit of detection. In both cases, the animals did not exhibit any clinical signs.

EEHV3-specific nucleic acids were detected in several trunk wash samples from E2. Positive results are shown in Table 2. No evidence of the presence of EEHV2 in the herd could be detected by qPCR.

## 4. Discussion

EEHV-HD has long been known and feared as a cause of death of Asian elephants. The risk for zoos that keep and breed African elephants was long considered to be low to negligible. Here, we report the death of an African elephant calf and the severe disease of a second animal in the same herd, which were most likely caused by EEHV6 infection. Our findings emphasize that EEHV monitoring and preparation for rapid therapeutic intervention in the event of illness are also crucial for African elephant husbandries. Like the course of disease described by Kongmakee et al. (2015) [7], only a few days passed between the onset of nonspecific clinical signs and the sudden and unexpected death of the elephant calf E1. The very rapid clinical course and the high case fatality rate are typical for primary EEHV infections in juvenile elephants and were also described by other authors [1,3,9]. The macroscopic and microscopic lesions observed here are in accordance with previously observed lesions after fatal EEHV-HD. Severe interstitial edema, hemorrhages, and changes in the endothelial cells and vessel walls were described in almost all necropsy reports [3,9,25,26,27,28]. A major macroscopic difference from previous necropsy reports was the absence of lingual cyanosis with accompanying edema. This finding was described both in clinically infected elephants [25,26,28] and at necropsy [9,25,26,27,28]. In their retrospective study, Perrin et al. (2021) stated that in eleven out of 24 cases presented with clinical abnormality of the tongue and 19 with post-mortem lingual cyanosis [25].

Mucosal hemorrhages have also been described in the literature in both Asian and African elephants [3,9,25,27,29]. Since the hemorrhages in the mucosa of the large intestine were predominantly found in proximity to the area with the large numbers of stones, they were probably caused by pressure. However, the EEHV infection could also have promoted bleeding.

The main difference in the histological findings was the absence of intranuclear inclusion bodies (INIBs) in all organ samples. There is little literature on postmortem studies and pathomorphological and pathohistological changes in African elephants with EEHV6 infections. However, in necropsy reports reviewed from Asian and African elephants, INIBs were generally described for all other genotypes of EEHV. They were observed in almost all parenchyma, but predominantly in endothelial cells [3,9,25,26,27,28]. Detecting INIBs in endothelial cells is also possible with immunohistochemical staining using von Willebrand factor [25]. Due to the lack of comparative literature, the question arises whether the lack of INIBs is a feature of EEHV6 infection or whether INIBs could not be visualized due to the severe endothelial damage in the presented case. Further investigations are therefore currently carried out to improve the detection of possible INIBs. However, it is not only possible to detect INIBs in endothelia cells. In their two papers, Richman et al. [26,28] also describe suspicious INIBs in epithelial cells in lymphoid follicles in a pulmonary nodule. They also detected INIBs in the stratum spinosum of skin nodules in an African elephant [28].

Whether rapid treatment with famciclovir prevented the fatal course of the disease (E2), as suggested in the case of a young animal in the USA that fell ill in 2009 and which had proven EEHV6 viremia [4], remains questionable. Serological analysis of blood samples taken at the time of E1’s death showed very low levels of EEHV-specific antibodies, which might explain the severe disease outcome [30]. In contrast, E2, the 8-year-old female, already had moderate levels of EEHV-specific antibodies when showing signs of disease, suggesting she had already been infected with at least one EEHV species in the past. Since the serological assay used in this study detects antibodies against multiple EEHV species, it currently remains unclear whether animal E2 developed symptoms after a primary EEHV6 infection or after viral reactivation. The animal was viremic for eight days and survived after five days of mild clinical disease and intense symptomatic and antiherpesviral treatment. Maximum viral loads of 1,8 × 10^7^ GE/mL plasma seem relatively high compared to recently published data [31] but can only be compared to a limited extent, as no viremia data are available for EEHV6, and PCR protocols vary. Whether pre-existing immunity, the rapid onset of intensive treatment, or both had a favorable influence on the course of the disease currently remains unclear. The incubation period of EEHV infections ranges between 7 and 14 days in Asian elephants [32]. The long interval between disease events observed here, as well as the presence of antibodies at the time of illness, could suggest an EEHV6 reactivation rather than an initial infection in E2. Nevertheless, it cannot be ruled out that E2 was only infected after a certain delay.

At the same time as E2, EEHV6 viremia was detected in E3. The viral loads determined here were only slightly above the detection limit of the qPCR. According to the literature, animals are considered at risk of EEHV-HD until they are 10 years old [6,7], but in the end, pre-existing immunity seems to be the decisive factor determining whether an animal dies, becomes seriously ill, or survives. In any case, data on EEHV6-HD is very limited. Therefore, older herd members should also be tested regularly as long as their immunity status remains unclear or insufficient. As expected, antibodies were also present in E3, and the animal remained free of clinical signs. In both cases, viremia was followed by trunk wash shedding for 18 days (E2) and 30 days (E3), which is comparable to other EEHV cases [31].

Stress is believed to be a relevant factor for herpesvirus reactivation. This was shown by Sylvester et al. (2024) [31] who monitored trunk wash shedding in the context of herd translocations. The EEHV6 shedding of E7 two weeks after the bull was introduced into the herd confirms previous findings of Sylvester et al. (2024) and shows the importance of these monitoring programs [31]. It is of high importance that elephant-keeping zoos are aware of viruses circulating in their herds.

Only EEHV6 was detectable in the organ samples of the deceased calf, and therefore monitoring of this EEHV species was implemented. To assess the presence and potential shedding of additional EEHV species, we retrospectively analyzed already available samples for the presence of EEHV2- and EEHV3-specific nucleic acids. Interestingly, seven trunk wash samples of E2, of which three were positive for EEHV6, were positive for EEHV3. Since positive trunk wash samples were not preceded by viremia, a reactivation of a latent EEHV3 infection can be assumed. Since E2 was born in Vienna, the primary infection must also have occurred in this herd. The clinical relevance of EEHV3 is as poorly understood as that of EEHV6. A case series documenting an EEHV3 outbreak at an American zoo underscores the potential risks, particularly to young animals [9]. In the outbreak described there, three viremic animals experienced mild symptoms, but tragically, two young African elephants succumbed to the virus, highlighting its potential severity and the threat it poses to juvenile elephants.

During the viremia of E2, different sample materials (PBMCs, plasma, whole blood) were repeatedly compared by EEHV6 qPCR. We received the lowest Ct (cycle threshold) values after collection and digestion of PBMCs from EDTA blood, followed by plasma and whole blood, which were about two to three Ct values higher. In the case of viremia, plasma seems to have no disadvantages compared to whole blood. However, the advantage of the faster analysis of plasma compared to PBMCs outweighs the slightly lower sensitivity. The occurrence of false-positive qPCR results, especially from blood samples of young elephants, should be avoided in any case.

## 5. Conclusions

EEHV-HD should be considered a serious threat regardless of the elephant species kept. Regular PCR tests of blood samples from individuals with unknown serological status, as well as trunk wash samples of all herd members, are strongly recommended. Additionally, the knowledge of serological status, especially of juveniles, is essential to perform reliable risk assessments for young individuals. Finally, a team of well-trained and medically equipped experts is pivotal to act quickly in the event of viremia and illness in order to minimize the risk of EEHV-related deaths in captive elephants.

## Figures and Tables

**Table 1:** PCRs and primer/probe sequences for the detection of EEHV6 DNA.

Virus/PCR	Primer Sequences (All Targeting POL Gene)	Reference	PCR Kit
EEHV (pan-PCR, semi-nested)	Pan-EEHV-1F/1nF (A1): 5′-ACAAACACGCTGTCRGTRTCYCCRTA-3′Pan-EEHV-1R (B1): 5′-GTATTTGATTTYGCNAGYYTGTAYCC-3′Pan-EEHV-nR (B2): 5′-TGYAAYGCCGTNTAYGGATTYACCGG-3′	[4]	a
EEHV-6 (nested PCR)	EEHV6_aF: 5′-GTGCCGAGTATAGCTTATCCG-3′EEHV6_aR: 5′-GCAGAATATTCGCGTGCATGC-3′EEHV6_nF: 5′-CATGGTCTATCTTACAGTCTACTAGC-3′EEHV6_nR: 5′-CTAGCATTATACAGGCATACAACCTG-3’	this study	
EEHV-6 (qPCR)	qEEHV-6-FW2: 5-GCATACAACCTGTGTTATTGTAC-3′qEEHV-6-RV: 5′-CGCTCTGCTAACCATGATGT-3′qEEHV-6-Probe: FAM-5′-ACCGATGACAATTTAACGTCACTTAGA-3‘-BHQ1	*	b

(a) QIAGEN Fast Cycling PCR Kit (Qiagen, Hilden, Germany); (b) Luna^®^ Universal Probe qPCR Master Mix (New England Biolabs GmbH, Frankfurt am Main, Germany); * Dr. J. Trimpert, Institute of Virology, Freie Universität Berlin; not published.

**Table 2:** EEHV3-positive trunk wash samples from E2.

Date of Sampling	Genome Equivalents Per Milliliter
31.08.2021	9.28 × 10^5^
20.09.2021	6.95 × 10^4^
02.11.2021	1.99 × 10^5^
20.07.2022	1.05 × 10^5^
17.10.2022	1.06 × 10^6^
19.10.2022	1.18 × 10^6^
19.12.2022	5.66 × 10^4^

## Data Availability

The raw data supporting the conclusions of this article will be made available by the authors upon request.

## Supplementary Materials

The following supporting information can be downloaded at https://www.mdpi.com/article/10.3390/ani15101482/s1. Figure S1: Comparism of EEHV6 qPCR by Dr. Trimpert, Freie Universität Berlin and by Stanton et al. 2012 [24]; Table S1: Results of EEHV6 qPCR in genome equivalents/mL; outbreak samples E2.

## Abbreviations

The following abbreviations are used in this manuscript:

EEHV	Elephant endotheliotropic herpesvirus
EDTA	Ethylenediaminetetraacetic acid
PCR	Polymerase chain reaction
qPCR	Quantitative polymerase chain reaction
PBS	Phosphate-buffered saline
BLAST	Basic Local Alignment Search Tool
BID	Lat.: bis in die (twice a day)
Ct	Cycle threshold
INIBs	Intranuclear inclusion bodies
